# Design and Implementation of an IoT-Based Low-Power Wearable EEG Sensing System for Home-Based Sleep Monitoring

**DOI:** 10.3390/s26061803

**Published:** 2026-03-12

**Authors:** Ya Wang, Jun-Bo Chen, Yu-Ting Chen

**Affiliations:** 1 School of Biomedical Engineering, South-Central Minzu University, Wuhan 430074, China; 2024120774@mail.scuec.edu.cn (Y.W.); 2022120691@mail.scuec.edu.cn (Y.-T.C.); 2 Key Laboratory of Cognitive Science, State Ethnic Affairs Commission, South-Central Minzu University, Wuhan 430074, China

**Keywords:** wearable EEG, sleep monitoring, instrumentation design, low-power electronics, Internet of Medical Things (IoMT)

## Abstract

Long-term home-based sleep monitoring requires wearable sensing devices that strictly balance signal precision with power constraints. This study presents the design and implementation of a low-noise, low-power wearable single-channel electroencephalography (EEG) system for automatic sleep staging. The hardware architecture integrates a TI ADS1298 analog front-end with an STM32F4 microcontroller, utilizing differential sampling and hardware-based filtering to effectively suppress power-line interference and baseline drift. System-level testing demonstrates an average power consumption of approximately 150.85 mW, enabling over 24.6 h of continuous operation on a 1000 mAh battery, which meets the requirements for overnight monitoring. To achieve accurate staging without draining the wearable’s battery, we adopted and deployed a lightweight deep learning model, SleePyCo, on the cloud backend. This architecture was specifically optimized for our edge–cloud collaborative execution, which combines contrastive representation learning with temporal dependency modeling. Validation on the ISRUC dataset yielded an overall accuracy of 79.3% ± 3.0%, with a notable F1-score of 88.3% for Deep Sleep (N3). Furthermore, practical field trials involving 10 healthy subjects verified the system’s engineering stability, achieving a valid data rate exceeding 97% and a Bluetooth packet loss rate of only 0.8%. These results confirm that the proposed hardware–software co-designed system provides a robust, energy-efficient IoMT sensing solution for daily sleep health management.

## 1. Introduction

Sleep quality and architecture are critical indicators of physiological and mental health, playing a vital role in the early screening and long-term management of chronic diseases [1,2]. Currently, Polysomnography (PSG) serves as the clinical gold standard for sleep assessment, utilizing multimodal signals such as electroencephalography (EEG), electrooculography (EOG), and electromyography (EMG). However, the complex instrumentation, high cost, and reliance on controlled laboratory settings render PSG unsuitable for long-term home monitoring [2,3]. Conversely, consumer wearables based on indirect signals (e.g., actigraphy or heart rate) offer convenience but often lack the signal fidelity required for accurate multi-stage sleep classification [3,4]. Meanwhile, non-contact technologies, such as optical fibers embedded in mattresses [5], offer unobtrusive monitoring alternatives, though wearable EEGs remain essential for capturing direct cortical activity.

To bridge the gap between clinical precision and home usability, portable EEG sensing nodes have emerged as a promising solution. However, designing a reliable single-channel EEG system for home use faces significant engineering challenges. First, clinical conventions (AASM) rely on complex signal features for staging (e.g., distinguishing N1 from REM), which is difficult to achieve with limited spatial information from a single channel [6]. Second, wearable instruments operate under strict power and size constraints, necessitating efficient hardware architectures to ensure continuous overnight operation without frequent recharging. Third, unlike controlled clinical environments, home settings introduce unpredictable noise and electrode contact variability, demanding robust analog front-end design and stable wireless transmission [7,8,9].

Addressing these challenges requires a holistic hardware–software co-design approach rather than focusing solely on algorithmic improvements [10]. Recently, the Internet of Medical Things (IoMT) and edge–cloud collaborative architectures have emerged as standard paradigms to resolve the inherent conflict between resource-constrained wearable nodes and computationally intensive health monitoring algorithms [11,12]. By offloading heavy data processing tasks to cloud servers, wearable devices can significantly extend battery life while still delivering real-time, clinical-grade analytical results [13]. In this study, we present the design and implementation of a low-power, wireless single-channel EEG sensor tailored for home-based sleep monitoring. The system integrates a high-precision ADS1298 analog-to-digital converter (ADC) with an STM32F4 microcontroller to achieve low-noise acquisition and real-time control. To enable accurate sleep staging within the computational limits of embedded platforms, we adopted and deployed a lightweight deep learning framework named SleePyCo [14]. This algorithm features a resource-efficient architecture combining contrastive pre-training with temporal context modeling, specifically optimized for single-channel signal processing. The main contributions of this work are summarized as follows:

(1) Design of a Low-Power Wearable EEG Sensing Node: We developed a compact sensor node featuring a highly integrated analog front-end and optimized power management. The system achieves an average power consumption of approximately 150.85 mW, supporting a theoretical battery life of 24.6 h on a standard 1000 mAh battery, ensuring reliability for full-night recording.

(2) Cloud-Based Inference in an IoT Architecture: We adopted and deployed a streamlined sleep staging algorithm (SleePyCo [14]) on the cloud server. By offloading the complex neural network computation to the cloud, the system successfully balances high classification accuracy with the strict low-power constraints of the wearable hardware.

(3) Field Validation and System Robustness: Beyond standard dataset evaluation, the instrument underwent practical validation through at-home trials involving 10 subjects. The results demonstrate engineering robustness, with a valid data transmission rate exceeding 85% and a Bluetooth packet loss rate of only 0.8%, confirming the system’s stability and practicality for daily health monitoring.

## 2. Materials and Methods

### 2.1. Hardware Design

**Figure 1:**
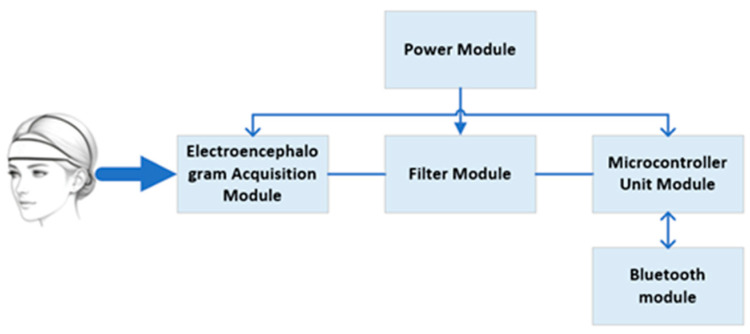
System hardware architecture diagram. The hardware modules include an EEG acquisition module, a filtering module, a power supply module, a microcontroller module, and a Bluetooth module.

**Figure 2:**
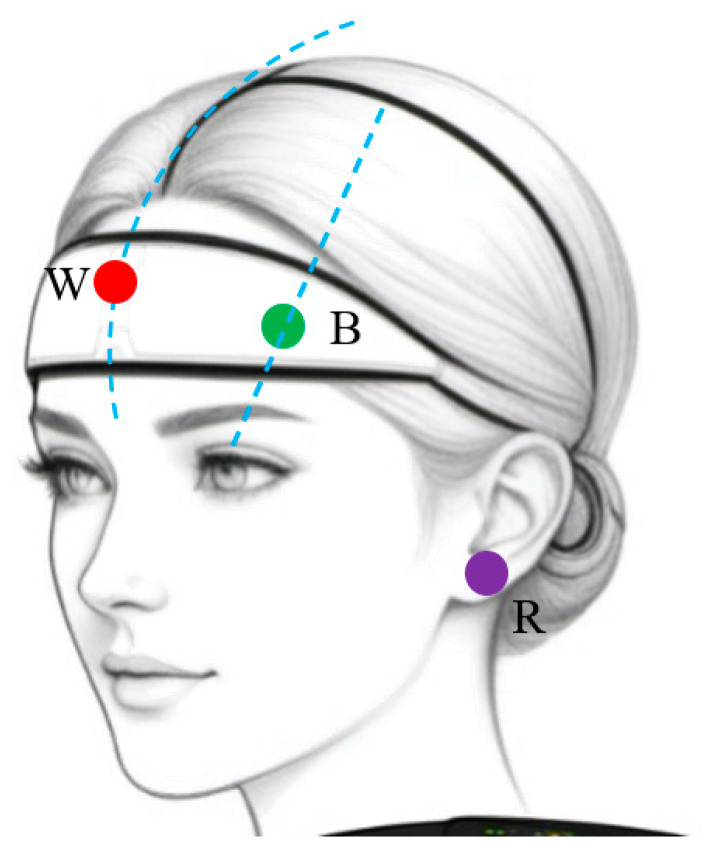
Electrode placement diagram. The system employs a single-channel differential montage: ‘W’ (Red) denotes the Working/Sensing electrode placed at the forehead; ‘B’ (Green) denotes the Bias/Reference electrode placed adjacently; and ‘R’ (Purple) denotes the Right-Leg Drive (RLD) electrode attached to the earlobe to suppress common-mode interference.

The hardware system is designed for low-noise, low-power EEG acquisition and integrates EEG acquisition, on-board signal processing, wireless transmission, and power management, as shown in Figure 1. The EEG acquisition module employs a TI ADS1298 analog front end with 8-channel 24-bit ΔΣ ADCs and programmable gain (1–24X), together with a built-in right-leg drive to suppress common-mode interference. Electrodes are arranged in a single-channel differential montage (sensing electrode at the forehead midpoint, reference at the earlobe) according to the 10–20 system, as illustrated in Figure 2. An external 0.5 Hz RC high-pass filter and a 50 Hz hardware notch filter are incorporated to attenuate baseline drift and mains interference [15].

Signal processing is performed by an STM32F429IGT6 microcontroller. The firmware, developed under Keil MDK-5, acquires raw data via SPI at 200 Hz, applies a 6th-order Butterworth 50 Hz IIR notch filter and a 4th-order Chebyshev Type-I 0.5–45 Hz band-pass filter, and then downsamples the signal to 100 Hz. The complete preprocessing pipeline for a 30 s epoch completes within 5 ms. Processed data are packetized using a custom binary protocol and transmitted via an HC-05 Bluetooth module (v2.0 + EDR), which operates in slave mode at 9600 bps with an effective range of approximately 10 m. To conserve power, the Bluetooth module is awakened by a GPIO only when data are ready and returns to sleep after transmission; transfers are scheduled at 5 s intervals. Since clinical sleep staging is performed on 30 s epochs, this 5 s micro-batch transmission introduces an acceptable maximum latency of 5 s without compromising the real-time monitoring experience. To prevent data loss while the Bluetooth module is in sleep mode, the STM32 SRAM acts as a local buffer for the continuous ADC samples. Data synchronization and integrity are strictly maintained by incorporating sequential packet IDs and timestamp offsets into the header of the custom binary protocol, allowing the mobile terminal to detect and request retransmission of any dropped packets.

The system is powered by a 3.7 V, 1000-mAh lithium battery. A PW5300 boost converter raises the voltage to 5 V, which is then regulated to 3.3 V (for the MCU and Bluetooth), +2.5 V, and −2.5 V (for the ADS1298) using AMS1117 and an LM2662 regulator, respectively. Battery charging and protection (overcharge, over-discharge, and short-circuit protection) are provided by a TP4056 charger IC.

### 2.2. Embedded Signal Processing and Classification Algorithm

While state-of-the-art deep learning models, such as large-scale convolutional networks and Transformers, have achieved remarkable accuracy in clinical sleep staging [16,17], their massive parameter counts and high computational demands make them unsuitable for low-latency IoT transmission and rapid edge–cloud inference [18]. Consequently, to address the non-stationary and multi-scale characteristics of single-channel EEG signals within our IoT architecture, this study adopts the lightweight sleep staging network, SleePyCo [14]. Originally proposed for robust EEG analysis, this framework was selected for our system because its compact parameter footprint ensures fast inference, low computational overhead, and minimal latency when deployed on the cloud backend. Rather than focusing on novel algorithmic architectural design, this study emphasizes the integration of this adopted model into our edge–cloud collaborative sleep monitoring system.

**Figure 3:**
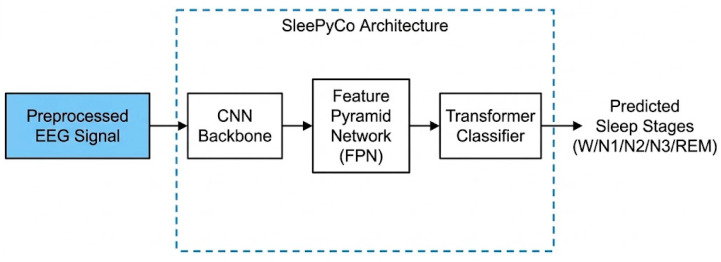
High-level block diagram of the adopted SleePyCo classification pipeline.

As illustrated in the high-level block diagram shown in Figure 3, the adopted staging algorithm consists of three primary modules: a 1D Convolutional Neural Network (CNN) backbone, lateral connections via a Feature Pyramid Network (FPN), and a Transformer-based classifier. The CNN backbone extracts both high-frequency fine details and lower-frequency global features. To effectively fuse these multi-scale features, the FPN aligns the temporal scales through lateral projections. Subsequently, a two-layer Transformer encoder is employed to model cross-epoch temporal dependencies using self-attention mechanisms, ultimately mapping the contextual features to the five distinct sleep stages.

For model preparation, we conducted offline training on a workstation using the ISRUC dataset, following the two-stage strategy (Contrastive Representation Learning and Multi-scale Temporal Context Learning) introduced by the original authors [14]. To make this paper self-contained, we briefly summarize the CRL strategy adopted from the original authors: during the pre-training stage, positive pairs are constructed by applying random data augmentations (such as scaling and shifting) to the same EEG epoch, whereas augmented views of different epochs within a mini-batch serve as negative pairs. The model is then optimized using the NT-Xent (Normalized Temperature-scaled Cross-Entropy) loss to pull positive pairs closer in the latent space while pushing negative pairs apart.

Once the optimal model weights were obtained, the network was frozen and deployed as a cloud inference service. In our system design (further detailed in Section 2.4), the resource-constrained STM32 microcontroller is dedicated exclusively to low-power signal acquisition, preprocessing, and Bluetooth transmission. The preprocessed EEG epochs are then securely forwarded via a mobile terminal to the cloud, where the SleePyCo engine performs real-time sleep staging. This edge–cloud collaborative approach not only maximizes the battery life of the wearable node but also guarantees high-accuracy classification using deep learning.

### 2.3. Evaluation Indicators

To evaluate the classification performance, standard metrics including Accuracy (ACC), Macro F1-Score (MF1), and Cohen’s Kappa (κ) were employed:

(1) Accuracy (ACC): quantifies the global classification correctness and is defined as:

(1) $$ACC=\frac{TP+TN}{TP+FP+TN+FN}$$

(2) Macro F1 Score (MF1): The average of the F1 scores for each class, used to evaluate the overall performance in multi-class classification. It is expressed as:

(2) $$MF1=\frac{1}{N_{c}}\sum_{j=1}^{N_{c}}{F1}_{j}=\frac{1}{N_{c}}\sum_{j=1}^{N_{c}}\frac{2\times {PR}_{j}\times {RE}_{j}}{{PR}_{j}+{RE}_{j}}$$

In Equation (2), ${PR}_{j}$ represents the precision of class j, and ${RE}_{j}$ denotes the recall of class j.

(3) Kappa coefficient (κ): assesses the level of agreement beyond chance. It is calculated as:

(3) $$\kappa =\frac{ACC-P_{e}}{1-P_{e}}=1-\frac{1-ACC}{1-P_{e}}$$

In Equation (3), $P_{e}$ represents the probability of the chance agreement.

### 2.4. System Integration and Application

**Figure 4:**
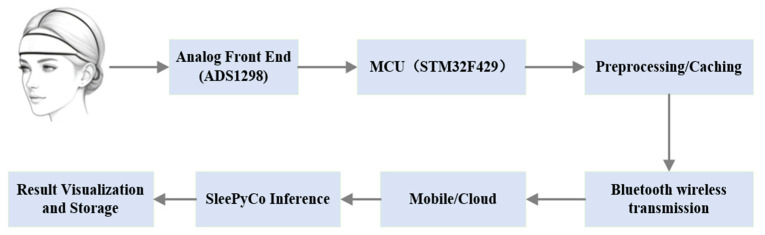
System Overall Operational Logic Diagram.

The system integration is designed to enable convenient at-home sleep monitoring through an end-to-end workflow encompassing hardware acquisition, wireless transmission, and mobile interaction, as illustrated in Figure 4. The wearable acquisition unit performs frontal differential EEG sampling, embedded bandpass and notch filtering, and short-term artifact marking. The preprocessed data are transmitted via Bluetooth to the mobile terminal using a structured framing protocol.

**Figure 5:**
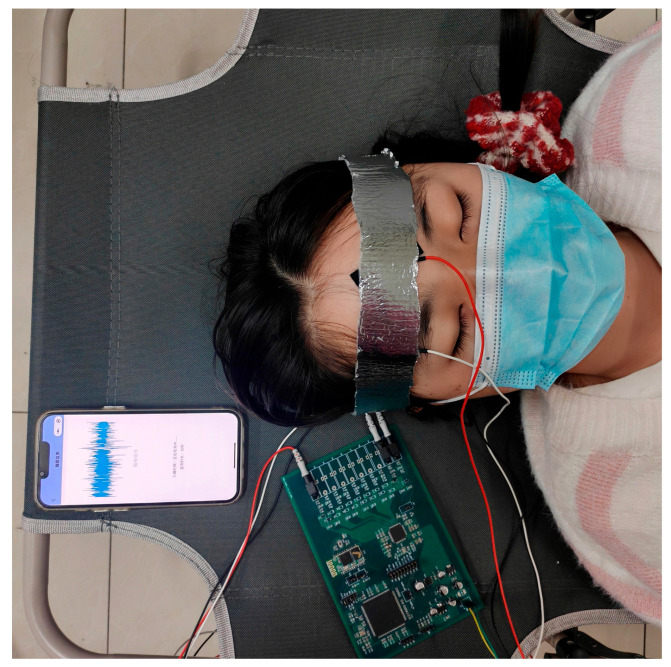
System overall test diagram.

The mobile application, implemented as a WeChat Mini Program, functions as the user interface layer, responsible for data reception, local caching, and preliminary unpacking. Batch or micro-batch data are then securely forwarded to a cloud inference service. The cloud-deployed SleePyCo model, running in a containerized environment, performs real-time sleep stage inference. The inference outputs undergo post-processing, including temporal smoothing and confidence evaluation, before being returned to the mobile device for visualization and long-term storage. The end-to-end workflow of the proposed system is illustrated in Figure 5.

### 2.5. Dataset and Signal Preprocessing

This section introduces the dataset and preprocessing steps used in the experiment for subsequent algorithm evaluation.

(1) Dataset

The ISRUC dataset is a publicly available sleep dataset provided by the International Sleep Research and Data Science Conference, primarily used in research on automated sleep staging and sleep quality assessment [19]. It encompasses EEG recordings from individuals across different ages and health statuses. Each recording contains multi-channel EEG signals, EOG signals, and EMG signals sampled at 200 Hz. All EEG signals were manually labeled by two experts according to AASM standards to indicate distinct sleep stages (e.g., awake, N1, N2, N3, and REM). For model evaluation, this study selected EEG data from the C4-A1 channel within the dataset. To ensure experimental accuracy, the data were used without additional processing and were analyzed exclusively using standard sleep stage data.

(2) Signal Preprocessing

To reduce noise and redundant information while balancing computational and hardware costs, the raw EEG signals in this study were subjected to anti-aliasing filtering before being downsampled to 100 Hz. This process preserves key sleep-related spectral components while reducing data rate and processing overhead [16,17]. Evaluation metrics included overall accuracy (ACC), Cohen’s κ (measuring annotation consistency), and the F1 score for each sleep stage, combining precision and recall. These metrics are widely adopted in sleep staging research to enable direct comparison with existing studies. To ensure label consistency and reproducibility, the annotations provided by Expert 1 in the ISRUC dataset were used as the reference labels for model training and evaluation.

## 3. Results

### 3.1. System Performance Evaluation

(1) Power Supply Test Results

The reliability of a wearable instrument depends fundamentally on its electrical stability and power efficiency. We conducted rigorous testing on the power management module using a precision oscilloscope. As shown in Table 1, the output voltage error across all rails remained below 1.2%, significantly outperforming the standard design requirement (<5%), which ensures a low-noise floor for the analog front-end.

(2) System Power Consumption Testing

In terms of energy efficiency, the system demonstrated an average power consumption of 150.9 mW during continuous operation. With a standard 1000-mAh lithium battery, the device achieves a theoretical runtime of 24.6 h, comfortably supporting full-night recording without interruption.

(3) Field performance.

Ten healthy volunteers (age 22–26 years; 4 males, 6 females; BMI 18.5–24.0) completed seven consecutive nights of at-home monitoring under ambient conditions of 22 ± 2 °C and 50 ± 5% relative humidity. Electrode sites were cleaned with 75% alcohol before each session and impedances were kept below 10 KΩ. Signal quality was verified via the mobile application (SNR ≥ 45 dB). Of 70 recorded nights, two sessions were excluded due to electrode detachment (valid data rate 97.1%). For the remaining 68 nights, the proportion of artifact-free epochs exceeded 85% and the mean SNR was 52 dB, demonstrating stable and robust home-based EEG acquisition.

### 3.2. Results on the ISRUC Dataset

K-fold cross-validation is a common model evaluation method. It involves dividing the dataset into k subsets, then sequentially using each subset as the test set while training the model on the remaining data k times. This validation method fully utilizes the dataset to provide a more reliable assessment of model performance. In this experiment, the k-fold cross-validation fold count for the ISRUC dataset is set to 10. On the ISRUC dataset, all cross-validation divisions are performed at the subject level to prevent epochs from the same subject appearing simultaneously in both training and test sets, thereby ensuring the independence and robustness of the evaluation.

Table 2 presents the classification performance using 10-fold cross-validation. The system achieved an overall accuracy (ACC) of 79.3% ± 3.0% (standard deviation) and a Cohen’s κ of 0.731, indicating substantial agreement with expert annotations. Notably, the model demonstrated superior performance in identifying Deep Sleep (Stage N3), achieving an F1-score of 88.3%. While the detection of transitional Stage N1 (F1 = 52.2%) remains challenging for single-channel configurations due to the lack of spatial information, the high precision in N3 and N2 stages is of greater clinical relevance for home-based health monitoring. These results confirm that the adopted lightweight model successfully balances computational efficiency with the classification accuracy required for assessing core sleep architecture.

### 3.3. Physiological Signal Verification in Real-World Scenarios

**Figure 6:**
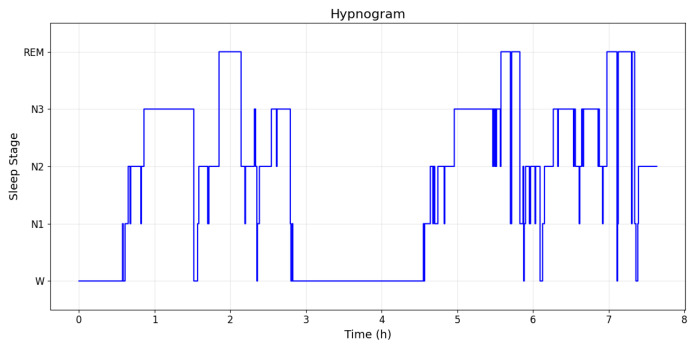
Sleep stage diagram for Subject I.

Continuously acquired EEG signals were segmented into 30 s epochs and used as inputs to the sleep staging model, enabling automatic classification of W, NREM (N1–N3), and REM stages. A representative hypnogram from Subject I is shown in Figure 6, illustrating a typical nocturnal sleep pattern. The horizontal axis represents time in epochs (120 epochs correspond to one hour), while the vertical axis indicates the corresponding sleep stages. The figure shows clearly delineated sleep cycles, each lasting approximately 90–110 min, consistent with patterns commonly observed in healthy adults. The proportions of total sleep time spent in each stage were as follows: W, 10.0%; N1, 9.2%; N2, 35.3%; N3, 38.7%; and REM, 16.8%. The proportion of N3 fell within the normal physiological range (20–40%), while that of REM was within the healthy standard range (15–25%).

**Figure 7:**
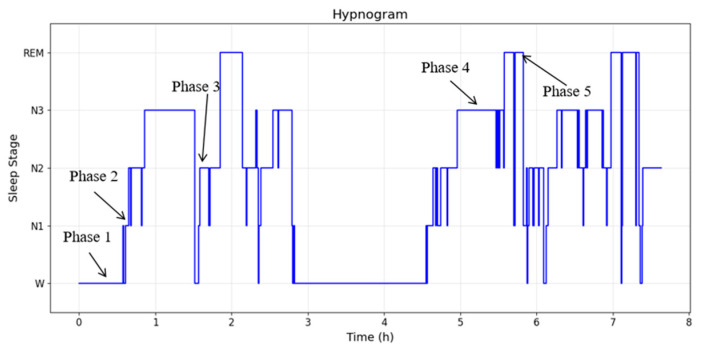
Randomly labeled stages of sleep stage diagram for Subject I.

To further evaluate the sleep staging accuracy, data from Test Subject I were selected for detailed analysis. Five distinct time periods were randomly identified and annotated on the hypnogram, as shown in Figure 7. By examining the corresponding EEG time-domain waveforms and spectral characteristics in each period, the model-predicted sleep stages were validated against their electrophysiological signatures.

**Figure 8:**
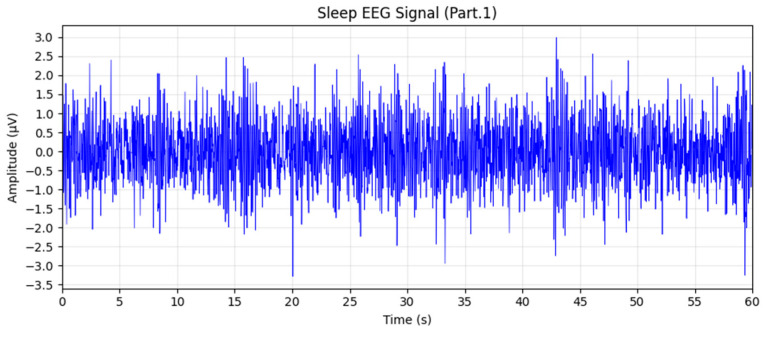
Sleep EEG waveform corresponding to Stage 1.

**Figure 9:**
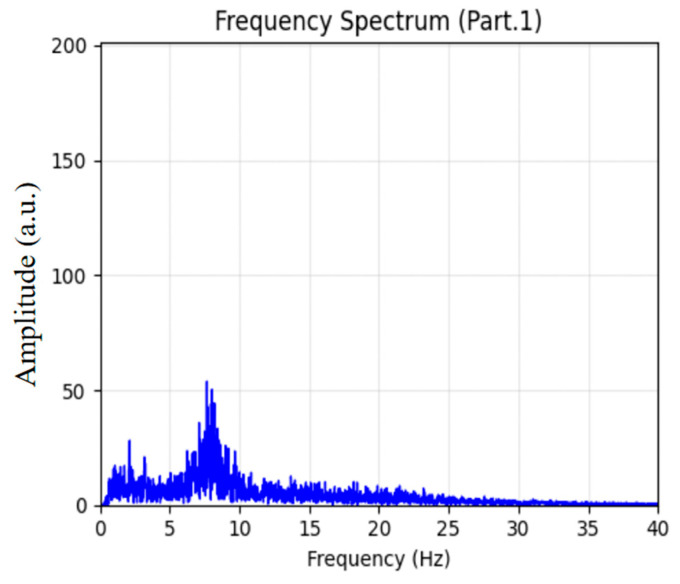
Sleep EEG spectrum analysis for Stage 1.

Two 60 s EEG segments were randomly selected from Stage 1. Their corresponding time-domain waveforms and spectral features are presented in Figure 8 and Figure 9, respectively. During this period, the EEG signals were dominated by low-amplitude α-wave activity, accounting for more than 50% of the total spectral power. This pattern is consistent with the EEG characteristics of wakefulness under eyes-closed resting conditions, confirming the reliability of the adopted model in accurately identifying the W stage.

**Figure 10:**
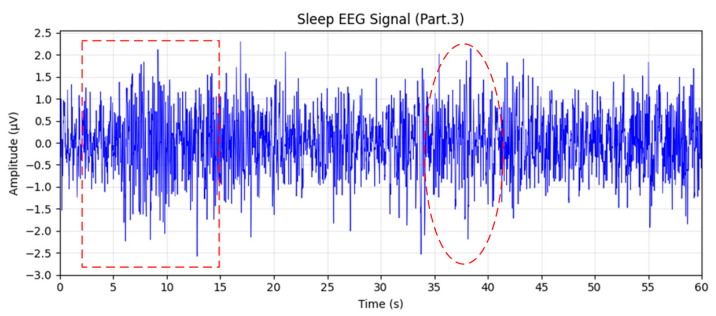
Sleep EEG waveform corresponding to Stage 3. The red dashed box indicates a sleep spindle, and the red dashed oval highlights a K-complex.

**Figure 11:**
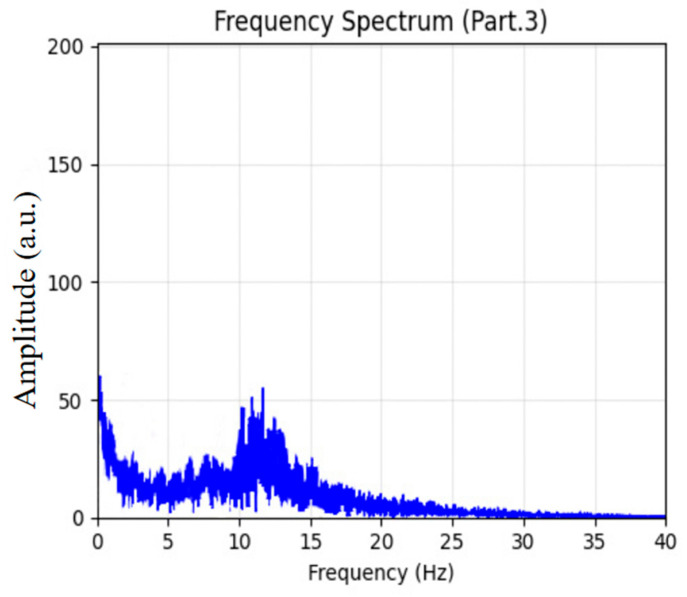
Sleep EEG spectrum analysis for Stage 3.

From Stage 3 (N2), two 60 s EEG epochs were randomly selected, as shown in Figure 10. The EEG signals exhibited significantly higher amplitudes than those in lighter sleep stages, with clear occurrences of sleep spindles (highlighted by red rectangles) and K-complexes (outlined with red dashed ellipses). The corresponding spectral analysis in Figure 11 revealed a pronounced peak within the 12–14 Hz spindle frequency band. These electrophysiological features are typical biomarkers of N2 sleep and validate the model’s robust classification performance for this stage.

**Figure 12:**
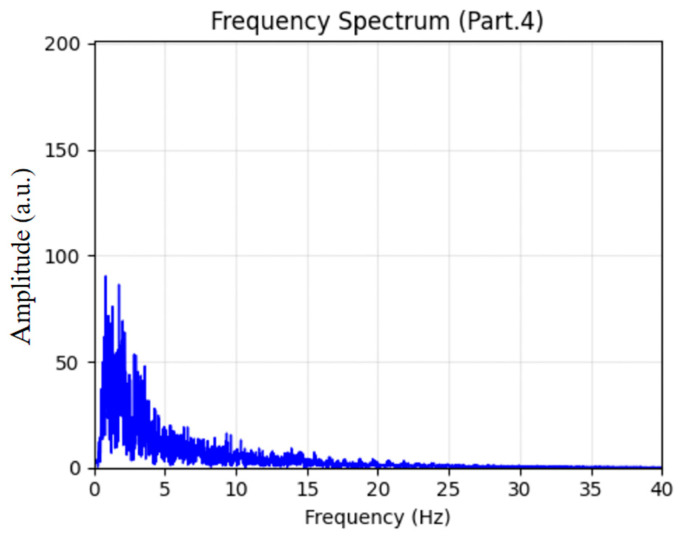
Sleep EEG spectrum analysis for Stage 4.

For Stage 4 (N3), the spectral characteristics are displayed in Figure 12. The power spectrum indicates dominant δ-band slow-wave activity (0.5–4 Hz), which aligns with the established diagnostic criteria for deep N3 sleep, thereby supporting the model’s accurate detection of this stage.

**Figure 13:**
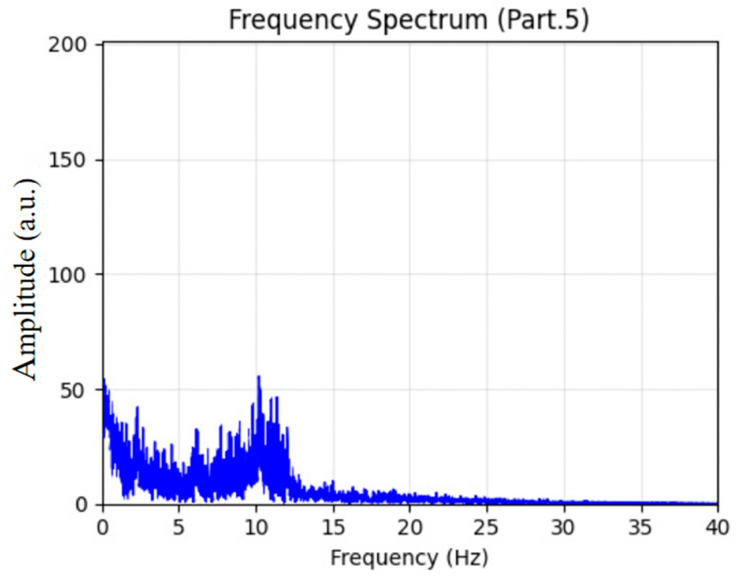
Sleep EEG spectrum analysis for Stage 5.

Similarly, Figure 13 illustrates the spectral characteristics for Stage 5 (REM). During this period, δ-wave power decreased while α-band activity re-emerged, forming a mixed α-θ pattern characteristic of REM sleep. These findings further confirm the physiological interpretability and staging reliability of the adopted model.

A quantitative analysis was also conducted to determine the temporal proportion of each sleep stage in the hypnograms of the subjects, with specific results shown in Table 3. The results indicate that the duration proportions of all sleep stages (N1, N2, N3 and REM) fell within normal physiological ranges.

In summary, the automated sleep staging model employed in this study demonstrates high staging accuracy and reliability, providing an effective technical reference for clinical sleep medicine research and at-home health monitoring.

## 4. Discussion

As summarized in Table 4, the proposed system achieves a competitive overall accuracy of 79.3% and a Cohen’s κ of 0.731 on the ISRUC-S1 dataset. While deep learning models running on high-performance servers (e.g., U-Sleep) may achieve marginally higher accuracy, our IoT-based solution is specifically designed for an edge–cloud collaborative architecture. By offloading the computational complexity of the deep learning algorithm to the cloud, we successfully maintained clinically relevant staging performance while allowing the resource-constrained wearable microcontroller to focus purely on low-power signal acquisition and wireless transmission.

A key strength of the instrument is its high sensitivity in detecting Deep Sleep (Stage N3), achieving an F1-score of 88.3%. Since N3 is the most restorative sleep stage and a critical indicator for physiological recovery, this high precision makes the device highly valuable for home-based health monitoring. Conversely, the recognition of the transitional Stage N1 remains a limitation (F1 = 52.2%). This is an inherent trade-off in single-channel EEG designs: distinguishing N1 from REM typically requires spatial information from multiple electrode sites (e.g., EOG), which would significantly increase hardware complexity and reduce wearer comfort [20]. From an engineering perspective, sacrificing N1 sensitivity for a compact, single-channel form factor is a justifiable compromise for long-term home usability. While theoretical approaches such as multimodal fusion [21] or temporal context modeling [22] could improve recognition, they impose hardware burdens that require careful system-level evaluation [23].

Hardware evaluation further validates the system’s practicality. The differential analog front-end effectively suppresses power-line interference, ensuring signal fidelity without bulky shielding. The average power consumption of 150.9 mW allows for >24 h of operation, addressing the “battery anxiety” common in wearable devices.

Despite the promising results, this study has several limitations that warrant future investigation. First, this study is limited by a relatively small sample size (10 healthy subjects). Additionally, the prolonged use of conventional wet electrodes during overnight recordings may lead to gel dehydration, potentially increasing skin-electrode impedance. Future iterations will explore flexible dry electrodes to enhance long-term wearing comfort [24] and maintain signal stability. Recent advancements in conductive polymer-based dry electrodes and microneedle arrays have demonstrated excellent potential in overcoming the limitations of traditional Ag/AgCl wet electrodes, offering stable skin-electrode contact impedance without the need for conductive gels [25,26]. Integrating these novel biocompatible materials into our IoMT sensing node will be a primary focus for our next-generation home-monitoring prototypes [27]. Second, while the algorithm’s accuracy was benchmarked on the ISRUC dataset (C4-A1 derivation), the actual wearable utilizes a frontal montage. Although frontal EEG effectively captures key sleep biomarkers (e.g., delta waves), future work will involve transfer learning on dedicated frontal datasets to further optimize real-world performance. Finally, the home-based field trials focused on demonstrating engineering feasibility and IoMT system robustness rather than clinical diagnostic accuracy, as concurrent PSG was not feasible in an uncontrolled setting. Future prospective studies will involve simultaneous clinical PSG recordings to thoroughly validate its diagnostic equivalence.

## 5. Conclusions

In this work, we designed and implemented a hardware-algorithm co-designed wearable EEG instrument for home-based sleep monitoring. Unlike purely algorithmic studies, we focused on the engineering challenges of low-power acquisition and embedded signal processing. The resulting system integrates a high-precision ADS1298 front-end with an STM32 microcontroller, achieving a low power consumption of 150.9 mW and a continuous runtime of 24.6 h, making it ideal for overnight recording.

To enable intelligent staging without draining the resource-constrained wearable hardware, we successfully adopted and deployed the pre-trained SleePyCo model on the cloud backend. Evaluation results show that the system provides reliable sleep staging accuracy (ACC = 79.3%, N3 F1 = 88.3%) while maintaining a low computational footprint. Furthermore, field trials with 10 participants demonstrated the instrument’s robustness in real-world environments, achieving a valid data transmission rate of over 97% via Bluetooth.

In summary, this study presents a practical, cost-effective engineering solution for daily sleep health management. Future work will explore the integration of dry electrodes and multimodal sensors (e.g., PPG) to further enhance wearer comfort and staging granularity.

## Figures and Tables

**Table 1:** Power Supply Test Results.

Voltage Source	Theoretical Voltage (V)	Measured Voltage Value (V)	Error
PW5300	5.00	4.99	0.2%
AMS1117_3.3	3.30	3.28	0.6%
AMS1117_2.5	2.50	2.49	0.4%
LM2662	−2.50	−2.47	1.2%

**Table 2:** Aggregated Confusion Matrix and Metrics per Class for Dataset C4-A1 (Right) Channel Obtained via 10-Fold Cross-Validation.

Predictive Classification							Performance Metrics (%)		
		W	N1	N2	N3	REM	PR	RE	F1
True Classification	W	16,459	1191	384	17	632	85.7	88.1	86.9
	N1	1919	5632	3280	37	1233	59.5	46.5	52.2
	N2	457	1691	24,523	1728	891	77.8	83.7	80.6
	N3	22	3	1853	14,163	68	88.8	87.9	88.3
	REM	353	952	1486	9	9062	76.2	76.4	76.3

**Table 3:** Number of Epochs and Percentage Distribution for Each Sleep Stage in Subject I.

Sleep Stages	N1	N2	N3	REM	Total
epoch number	57	218	239	104	618
Duration Distribution	9.2%	35.3%	38.7%	16.8%	100%

**Table 4:** Comparison of the Proposed System with State-of-the-Art Staging Methods on the ISRUC-S1 Dataset.

Study	EEG Epoch	Per-Class-F1					Overall	
		W	N1	N2	N3	REM	ACC	MF1
DeepSleepNet	25	81.55	38.25	68.90	81.17	62.17	69.84	66.41
IITNet	10	84.60	40.51	78.39	85.27	79.15	77.89	73.59
U-Sleep	35	86.34	44.16	79.07	85.38	81.48	78.89	75.29
Attn Sleep	1	84.54	42.41	75.81	83.45	69.92	75.72	71.23
SleepExpertNet	20	72.59	14.25	66.61	72.69	58.05	64.89	56.84
NeuroNet-B(TCM)	20	84.50	46.09	77.25	86.86	72.57	77.05	73.45
SleePyCo	30	86.9	52.2	80.6	88.3	76.3	79.3	76.9

## Data Availability

The original data presented in the study are openly available in ISRUC at https://sleeptight.isr.uc.pt/ (accessed on 3 February 2026).
